# Role of Nrf2 in Parkinson’s Disease: Toward New Perspectives

**DOI:** 10.3389/fphar.2022.919233

**Published:** 2022-06-24

**Authors:** Xin-xing Yang, Rong Yang, Feng Zhang

**Affiliations:** ^1^ Laboratory Animal Center and Key Laboratory of Basic Pharmacology of Ministry of Education and Joint International Research Laboratory of Ethnomedicine of Ministry of Education and Key Laboratory of Basic Pharmacology of Guizhou Province, Zunyi Medical University, Zunyi, China; ^2^ Collaborative Innovation Center of Tissue Damage Repair and Regeneration Medicine of Zunyi Medical University, Zunyi, China

**Keywords:** Parkinson’s disease, pathogenesis, Nrf2, neuroprotection, oxidative stress

## Abstract

Parkinson’s disease (PD) is one of the most common and chronic degenerative diseases in the central nervous system. The main pathology of PD formation is the progressive loss of dopaminergic neurons in substantia nigra and the formation of α-synuclein-rich Lewy bodies. The pathogenesis of PD is not caused by any single independent factor. The diversity of these independent factors of PD, such as iron accumulation, oxidative stress, neuroinflammation, mitochondrial dysfunction, age, environment, and heredity, makes the research progress of PD slow. Nrf2 has been well-known to be closely associated with the pathogenesis of PD and could regulate these induced factors development. Nrf2 activation could protect dopaminergic neurons and slow down the progression of PD. This review summarized the role of Nrf2 pathway on the pathogenesis of PD. Regulation of Nrf2 pathway might be one of the promising strategies to prevent and treat PD.

## Introduction

### Overview and Current Situation of Parkinson’s Disease

Parkinson’s disease (PD), known as tremor palsy, is a group of neurodegenerative diseases with a long pathological cycle. The clinical manifestations of PD are dominated by movement disorders with early resting tremor, myotonia, and postural abnormalities (De Virgilio et al., 2016). These manifestations are mainly related to a deficiency of dopamine in the posterior shell nucleus and motor circuits. In addition, PD nonmotor disorder symptoms include olfactory disturbances, depression, dementia, and anxiety (Molina Ruiz et al., 2016). At present, PD occurs in the middle and old age. According to the statistics from World Health Organization (WHO), the incidence rate of PD has a significant upward trend. The proportion of the world’s population over 60 by 2050 will be more than nearly double by 2015, from 12% to 22%, while the incidence rate is about 0.5% at 50 years old and up to 4% at 80 years old (Deng et al., 2013).

Pathological features of PD are accompanied by a continuous decrease in dopaminergic neurons containing neuromelanin in the substantia nigra and the formation of α-synuclein-rich Lewy bodies (Hustad & Aasly, 2020). Importantly, the pathogenesis of PD may arise from multifactorial effects, especially on iron aggregation, oxidative stress, neuroinflammation, mitochondrial dysfunction, age, and environmental and genetic alterations. The diversity of the interaction of these factors with PD progression makes their pathogenesis of PD more complex.

### Overview of Nrf2

**FIGURE 1:**
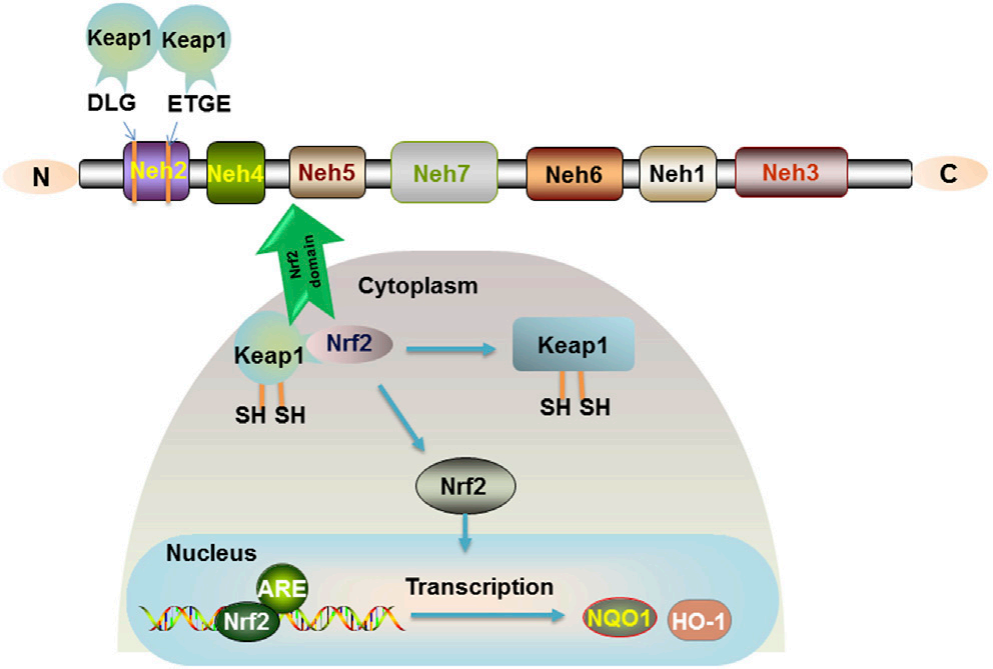
Nrf2 signaling pathway. The relative positions of the Neh domains of transcription factor Nrf2 are shown. The Neh2 structural domain containing multiple lysine residues at the N terminus is present with a high affinity of ETGE motif and a lower affinity of DLG motif. Keap1 is a sparse group-rich protein that inhibits Nrf2. Keap1 degrades Nrf2 through polyubiquitination and phosphorylation modification of Nrf2. In the organism, Nrf2 is bound to the cytoplasm by Keap1, and only when the cysteine residues of Keap1 are modified to cause conformational changes, Nrf2 is released into the nucleus and binds to the ARE to promote the expression of the target genes NQO1 and HO-1.

Nuclear factor-erythroid 2-related factor 2 (Nrf2), a protein with 605 amino acids, was cloned in 1994. It is a member of the Cap ‘n' Collar (CNC) family of transcription factors and contains a basic leucine zipper (bZIP). Nrf2 is composed of six highly conserved structural domains (Neh1-6) (Li et al., 2020). The Neh1 domain is a key bridge in the formation of dimerization of Nrf2 with DNA and responsible for the dimerization of broad-complex, tram track, and bric-abrac (BTB) and CNC homolog (Bach) proteins. The Neh2 structural domain containing multiple lysine residues at the N terminus is present with a high affinity ETGE motif and a lower affinity DLG motif (Tong et al., 2006). A Neh2 with two motifs will bind two Kelch-like ECH-associated protein 1 (Keap1), and Nrf2 is ubiquitinated when they bind to form a stable complex (Figure 1). C-terminal Neh3 maintains the transcriptional activity of Nrf2, and the two acidic residue-rich Neh4 and Neh5 interact with CREB-binding protein (CBP) and transcriptional coactivators. Neh6, which contains a large number of serine residues, has two motifs, DSGIS and DSAPGS, and is associated with glycogen synthase kinase-3 (GSK-3) phosphorylation. However, the Neh7 structural domain of Nrf2, which is an inhibitory domain, was reported in the recent literature. Neh7 affects Nrf2 activity by inhibiting the RXRα receptor and then allowing Nrf2 to block the binding of transcriptional coactivators to the Neh4 and Neh5 structural domains (Zgorzynska et al., 2021).

Importantly, Nrf2 is a key factor in antioxidative stress and mediates oxidative stress and immune inflammation. Under normal conditions, Nrf2 is short-lived and induces ubiquitination and proteasomal degradation of Nrf2 in the cytoplasm by acting with the negative regulator Keap-1 (Canning et al., 2015). In response to oxidative stress or electrophilic substances, Keap-1 is modified and Nrf2 is activated into the nucleus, where the Neh1 domain of the CNC protein Nrf2 in the nucleus forms a dimer with small Maf proteins (sMafs) that recognize and bind to antioxidant response elements (ARE) (Niu et al., 2021), further binding to the ARE sequence and activating transcription of cytoprotective factors. Regulation of the Keap1-Nrf2-ARE signaling pathway has been confirmed to generate potential beneficial effects (Figure 1).

## Key Influencing Factors and Pathological Mechanisms of PD

The etiology of PD has been difficult to elucidate, which appears to be related to a complex interplay of multiple factors. These factors include iron aggregation, oxidative stress, neuroinflammation, mitochondrial dysfunction, age, environment, and genetics.

### Iron Death

Trace metals are essential elements for life and usually present in low concentrations. In case of the brain, its demand for iron is relatively high, such as nutrient and oxygen transport, which requires the complementary involvement of iron. Fe^3+^ is pooled with the iron-binding protein transferrin, in which Fe^2+^ is involved in regulation as a cofactor for several enzymes (Mills et al., 2010). Disruption of the regulatory balance of micronutrients may lead to central nervous system (CNS) impairment and the development of diseases, such as Alzheimer’s disease (AD) and PD. If iron levels are not controlled, normal iron transport is interrupted and excess iron produces reactive oxygen species (ROS) and immune inflammation. Cellular iron acquisition involves the non-transferrin-bound iron uptake (NTBI) pathway and the classical transferrin-bound iron (TBI) uptake pathway (McCarthy & Kosman, 2015). Blockage of iron metabolic pathways would result in abnormal increases or decreases in iron and then cause disease.

The term “iron death” was introduced in 2012 and iron death is a novel cell death specifically associated with iron, which differs from programmed cell death (Dixon et al., 2012). It occurs through a vicious cycle of metal-protein binding followed by induction of aggregation formation through disruption of mitochondrial function, which depletes adenosine triphosphate and induces cell death through mechanisms, such as apoptosis and necrosis. It can be regulated by glutathione (GSH) peroxidase 4 GPx4. In 2018, the Nomenclature Committee on Cell Death (NCCD) defined “iron death” as a form of regulated cell death (RCD), caused by GPx4 constitutively controlled by oxidative alterations in the intracellular microenvironment, which can be inhibited by iron chelators and lipophilic antioxidants (Galluzzi et al., 2018). Also, iron death is regulated by several cellular metabolisms (iron metabolism, amino acid metabolism, lipid metabolism, and redox homeostasis) (Jiang et al., 2021). The programmed cell death plays an integral role in various aspects of growth and development.

As a cofactor of tyrosine hydroxylase, iron participates in the regulation of dopamine generation in the brain. Iron contributes to the properties of oxidants and may induce oxidative stress by producing ROS through Fenton reaction and Hubble Weiss reaction. It further releases iron from iron-containing proteins, such as ferritin, hemoglobin, and iron-sulfur clusters, creating a vicious positive feedback loop that exacerbates the toxic effects of iron overload in the brain (Mezzaroba et al., 2019). In patients with PD, the dysregulation of iron metabolism promoting PD deterioration was demonstrated using magnetic resonance imaging (MRI) and spectroscopy (MRS) analysis.

### Oxidative Stress

**FIGURE 2:**
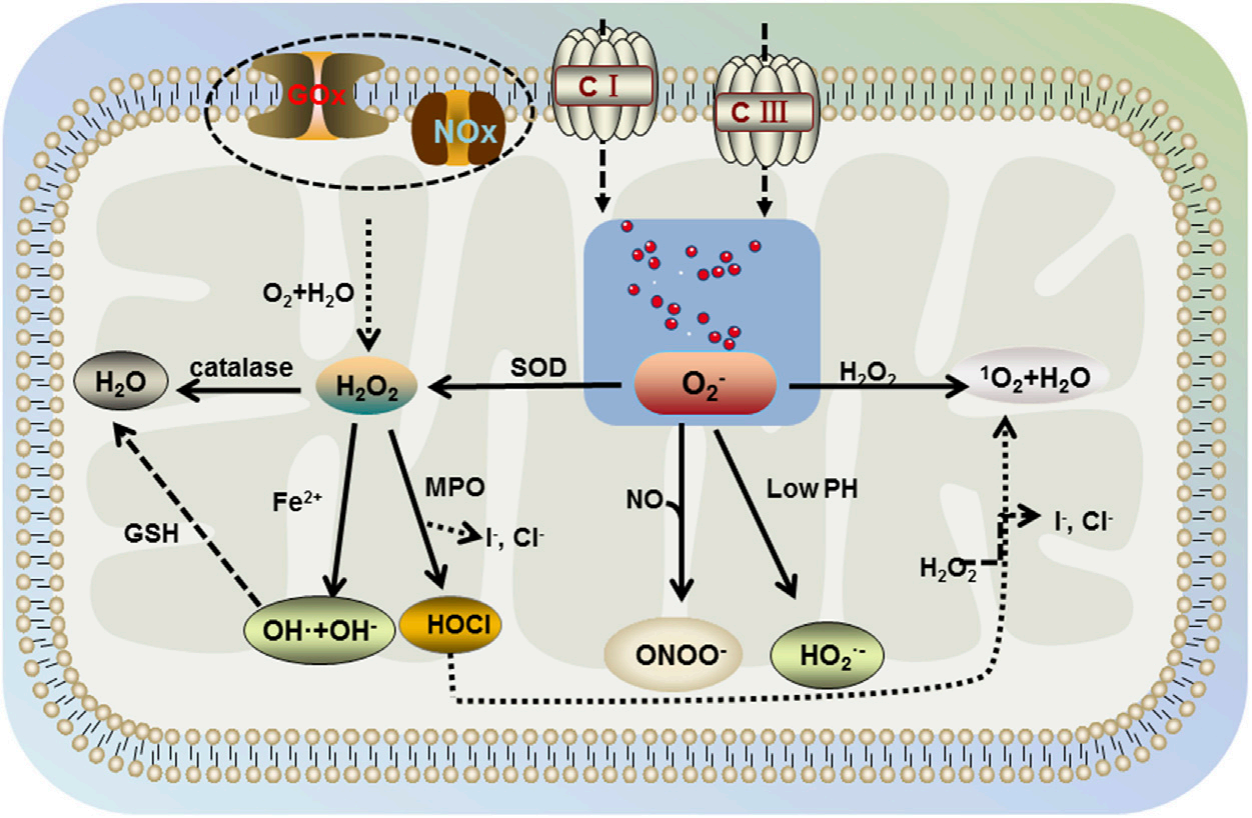
Generation of ROS. The aerobic metabolism of cells is accompanied by the generation of ROS including nonradicals hypochlorous acid (HOCl), singlet oxygen, hydrogen peroxide (H_2_O_2_), and free radicals, such as superoxide anion (O_2_^−^), superoxide, and hydroxyl radicals. Mitochondria contain large amounts of water and O_2_, which produce H_2_O_2_ intracellularly under the action of glucose oxidase (GOx) and NADPH oxidase (NOx). H_2_O_2_ further decomposes into water, superoxide, hydroxyl radicals, and hypochlorous acid in the environment involving catalase, Fe^2+^, and myeloperoxidase (MPO). Then, hydroxyl radical generates water under the action of glutathione (GSH), hypochlorous acid further generates singlet oxygen and water in the presence of H_2_O_2_. Free oxygen in mitochondria would be generated by superoxide anion under the action of mitochondrial complex I and complex III, which interacts with H_2_O_2_ in the body to produce singlet oxygen and water (same as hypochlorite). In addition, superoxide dismutase (SOD) catalyzes the decomposition of superoxide anion into H_2_O_2_, which further generates the benign product water. However, under acidic conditions (lower pH), superoxide anion is protonated and under nitric oxide conditions, peroxynitrite occurs.

In the human body, cells can use oxygen to participate in a range of detoxification and energy-producing reactions. Upon the redox reactions in organelles, such as mitochondria, peroxisomes, and endoplasmic reticulum (Sies, 2020), the aerobic metabolism of cells is accompanied by the generation of ROS, including nonradicals hypochlorous acid (HOCl), monoclinic oxygen, hydrogen peroxide (H_2_O_2_), and free radicals, such as superoxide anion (O_2_^−^), nitric oxide, superoxide, and hydroxyl radicals (Figure 2) (Bedard & Krause, 2007; D'Autréaux & Toledano, 2007; Siti et al., 2015). The nature of ROS determines its susceptibility to react with other reducing molecules and play an irreplaceable role in the mitochondrial respiratory chain. In the normal organism, ROS are beneficial. However, when the oxidative nature of ROS is greater than the antioxidant regulation of the organism, it is accompanied by the occurrence of oxidative stress. Excess ROS react with intracellular macromolecules, leading to different degrees of structural and functional changes in cells, which in turn cause physiological pathologies (Li et al., 2020). Compared to other organs, brain is the most active in oxidative metabolism and has a high demand for oxygen, with its oxygen consumption accounting for 20% of the body (Niu et al., 2021). At the same time, brain phospholipids contain polyunsaturated fatty acids, and their weak antioxidant properties and rich lipid content make their cells susceptible to oxidative damage. Dysregulated mechanisms of ROS increase susceptibility to damage and functional defects in neuronal cells, which is closely associated with the development and progression of neurodegenerative diseases, such as PD.

In addition, oxidative stress results from an imbalance in the body’s metabolism due to an excess of reactive substances, such as ROS. Oxidative stress affects cellular functions by targeting macromolecules (nucleic acids, lipids, and proteins) and further induces dopamine neuron loss and thus leads to PD. Lipid peroxidation is a prominent manifestation of oxidative stress damage to neurons, which causes structural damage to cell membranes, leading to apoptosis and necrosis as the integrity of cell membrane function is disrupted (Bae et al., 2013). Inhibition of lipid peroxidation effectively prevented apoptosis, highlighting a novel mechanism underlying dopamine neuronal death caused by lipid peroxidation in the brain (Angelova et al., 2020). The relevance of oxidative stress to PD is also reflected in changes in familial risk factors for PD, where various genes are involved in the occurrence of oxidative stress, such as α-synuclein (SNCA), parkin RBR E3 ubiquitin protein ligase gene (PRKN), PTEN-inducible putative kinase 1 gene (PINK1), Parkinson’s disease-associated deglycosylase gene (DJ-1), Leucine-rich repeat kinase 2 gene (LRRK2), F-box protein 7 gene (FBXO7) and ATPase 13A2 gene (ATP13A2) (Chang & Chen, 2020; Dorszewska et al., 2021). The SNCA gene is encoded by α-synuclein and its variation is closely associated with the development of familial PD. The deglycosylase activity of DJ-1 reduces the glycosylation and aggregation of α-synuclein in dopamine neurons (Sharma et al., 2019). LRRK2 has been linked to the biology of microglia, where LRRK2 may contribute to PD pathogenesis by altering oxidative stress signaling (Russo et al., 2019), suggesting that LRRK2 is highly involved in oxidative stress-related pathways. PRKN, PINK1, FBXO7, and ATP13A2 are associated with autosomal inheritance of PD. The point mutation loci related to PD are, respectively, PARK2, PARK6, PARK15, and PARK9.

### Neuroinflammation

Neuroinflammation is the basic immune response in CNS. Neuroinflammation at normal physiological concentrations protects neurons from damage and neuroinflammation acts in reverse if the organism’s normal values are exceeded. Although neuroinflammation is not specific to PD, it is one of the hallmarks of PD pathophysiology (Kaur et al., 2017).

The activation of glial cells (including microglia and astrocytes) is closely associated with neuroinflammation. In turn, infiltration and reactivation of T cells may exacerbate neuroinflammation triggering elevated levels of neurotoxicity (Kustrimovic et al., 2019). When microglia are in the activated state, their morphology becomes phagocytic and major histocompatibility complex (MHC) antigens are induced to be expressed and proinflammatory cytokines are released. In neuroinflammatory response, microglia activation, regulated by cyclooxygenase-2 (COX-2), toll like receptors 2 (TLR2) and toll like receptors 4 (TLR4) (Kaur et al., 2017), is of particular interest in PD. Microglial COX-2 expression was highly increased in PD patients. The lack of negative regulation of CD200-CD200R signaling also increased microglia activation and exacerbated the degeneration of dopamine neurons in experimental PD animal models. In PD, the aggregated ubiquitinated proteins following neuronal injury activated microglia, allowing for the production of proinflammatory mediators, such as prostaglandin E2 (PGE2) and prostaglandin A1 (PGA1). Increased expressions of cytokines, such as tumor necrosis factor-α (TNF-α), interleukin-1β (IL-1β) and interleukin-6 (IL-6), and transforming growth factor-β1 (TGF-β1), have been observed in PD (Boka et al., 1994; Allen Reish and Standaert, 2015). The release of these toxic substances from microglia might be involved in the death of dopamine neurons.

In addition, astrocytes have an important role in the regulation of immune responses in PD. Like microglia, astrocytes produce inflammatory factors that have an important impact on PD development. Astrocyte and microglia hyperplasia can result from overexpression of α-synuclein. Activation of LRRK2 by α-synuclein released from neurons in microglia *via* TLR2. Nuclear factor of activated T cells 2 (NFATc2) is directly phosphorylated as a substrate of LRRK2 kinase, and NFATc2 accelerates its translocation to the nucleus. Regulation of LRKK2-NFATc2 signaling cascade might identify new targets in the treatment of PD (Wang G et al., 2021). The level of inflammatory cytokines released by astrocytes is influenced by the dopamine receptor D2 (D2R). The reduction of astrocyte D2R in the aging brain has an important impact on immune homeostasis and ultimately contributes to the progression of PD.

### Mitochondrial Dysfunction

Although the exact pathogenesis of PD is complex, mitochondrial dysfunction is verified to be prominent in the development of PD. Mitochondria serve as a major source of ROS production. Since there is an imbalance between ROS production and consumption, the generation of oxidative stress can compromise mitochondrial function, and apoptosis or necrosis may then lead to dopamine neuronal loss in PD *via* a mitochondria-dependent pathway. The pathogenic mechanisms of mitochondria include defective mitochondrial biogenesis, mitochondrial DNA (mtDNA) mutations in mitochondria and environmental and genetic factors leading to PD. In addition to mitochondrial autophagy, altered mitochondrial dynamics and abnormal Ca^2+^ homeostasis are also closely related to PD pathology (Rani & Mondal, 2020). The electron transport chain (ETC) is the basis of mitochondrial energy production and its energy plays a key role in neuronal activity. PD-associated mutant genes PRKN, PINK1, and DJ-1 are directly involved in mitochondrial function, and a part of LRRK2 and glucocerebrosidase 1 (GBA1) is shown to indirectly affect the respiratory chain. Neurotoxins, such as rotenone, could disrupt mitochondria-associated membranes (MAMs) by altering Ca^2+^ homeostasis in mitochondria (Lee et al., 2018; Cherubini et al., 2020). Furthermore, mitochondrial dysfunction plays a critical role in the progression of PD by causing a series of alterations in misfolded proteins.

### Aging, Environment and Genetics

Environmental factors and genetic variants can produce oxidative stress that may lead to loss of dopamine neurons (Delamarre & Meissner, 2017; Vázquez-Vélez & Zoghbi, 2021). In addition, aging is the most important risk factor for PD, resulting in changes of body function that make PD more sensitive to abnormal manifestations in the brain. The increased aging is undoubtedly further aggravating the incidence of PD. Mutations in the glucocerebrosidase (GBA) gene, responsible for encoding the lysosomal enzyme, are the most common genetic risk factors that promote the progression to PD (Riboldi & Di Fonzo, 2019). Common autosomal genes (Kim et al., 2017; Fais et al., 2021) are shown in Table 1. Studies have shown that 5–10% of familial inherited PD is mediated by related genes, such as LRRK2, PINK1, and DJ-1 (Bonam & Muller, 2020). In recent years, transposable elements (TEs), long considered as “junk” DNA, have gained attention as pathogenic factors in PD. TE is a key pathogenic link between brain aging and neurodegeneration. Activation of TEs could be observed in most human diseases. Preliminary sequencing of the human genome revealed that approximately 45% of human DNA is derived from TEs (Ravel-Godreuil et al., 2021).

**TABLE 1:** Common autosomal genes and PD sites.

	Genes	Acts on PD sites
Autosomal dominant [39, 40]	SNCA	PARK1/PARK4
	Chromosome 2p13 gene	PARK3
	Ubiquitin C-terminal hydrolase L1 gene (UCHL1)	PARK5
	LRRK2	PARK8
	GRB10 interacting GYF protein 2 gene (GIGYF2)	PARK11
	Htra serine peptidase 2 gene (HTRA2)	PARK13
	Retrotransposon complex component gene (VPS35)	PARK17
	Eukaryotic translation initiation factor 4 γ 1 gene (EIF4G1)	PARK18
	Transmembrane protein 230 gene (TMEM230)	PARK21
	Curly helix structural domain (CHCHD2), RIC3 acetylcholine receptor chaperone gene (RIC3)	PARK22
Autosomal recessive	PRKN	PARK2
	PINK1	PARK6
	DJ-1	PARK7
	ATP13A2	PARK9
	Phospholipase A2 group VI gene (PLA2G6)	PARK14
	FBXO7	PARK15
	DnaJ heat shock protein family (Hsp40) member C6 gene (DNAJC6)	PARK19
	Synapsin 1 gene (SYNJ1)	PARK20
	Vesicular protein sorting 13 homolog C gene (VPS13C)	PARK23
X Genetics	PARK12	PARK12
Unknown Genetic	None	PARK10/PARK16

Environmental pollution is also a risk factor for the development of PD, including the pesticides paraquat (PQ) or maneb/mancozeb, rotenone (inhibits mitochondrial complex I), organochlorines, and organophosphates. Metals, such as iron and manganese, also play an important role in PD. By evaluating long-term exposure to air pollution and PD, it was found that nitrogen dioxide in the air or long-term exposure to PM2.5 increased the risk of developing PD and had a high incidence (Jo et al., 2021). Air pollution may increase the risk of developing PD through a variety of potential mechanisms, including direct neuronal cytotoxicity, induction of systemic inflammation indirectly promoting CNS inflammation, deficiency of antioxidant enzyme activity in the organism, and alterations in gut colony physiology and microbiome. Understanding how environmental exposures affect the pathogenesis of PD is a landmark for reducing the incidence of the disease and finding disease remission therapies (Murata et al., 2022). Viral infections can also affect PD, such as coronavirus disease 2019 (COVID-19) and severe acute respiratory distress syndrome coronavirus 2 (SARS-CoV-2) infections. Expression pathways, such as oxidative stress, organismal inflammation, and protein biomolecule aggregation have fundamental commonalities in PD and COVID-19 disease. SARS-CoV-2 infection is not common in patients with PD, but PD mortality has been indicated to be closely associated not only with older age, but also with a longer disease duration and multiple mechanisms. For example, viral interactions with the brain dopaminergic nervous system affect PD through systemic inflammatory responses and alterations in the gut microbiome (Rosen et al., 2021).

Additionally, three million gut microbiota (GM) are identical in about 1/3 of the population comparisons, and GM is 150 times larger than the human genome. The number of Prevotella has a significant reduction in fecal samples from PD patients, and GM of different bacterial families is strongly associated with good or bad motor function in PD patients (Scheperjans et al., 2015). GM is important in regulating the homeostasis of the organism and also can be involved in neurodegenerative diseases by interconducting with neurons in the brain *via* direct autonomic nervous system and immune and neuroendocrine pathways.

## Role of Nrf2 Pathway on PD

**FIGURE 3:**
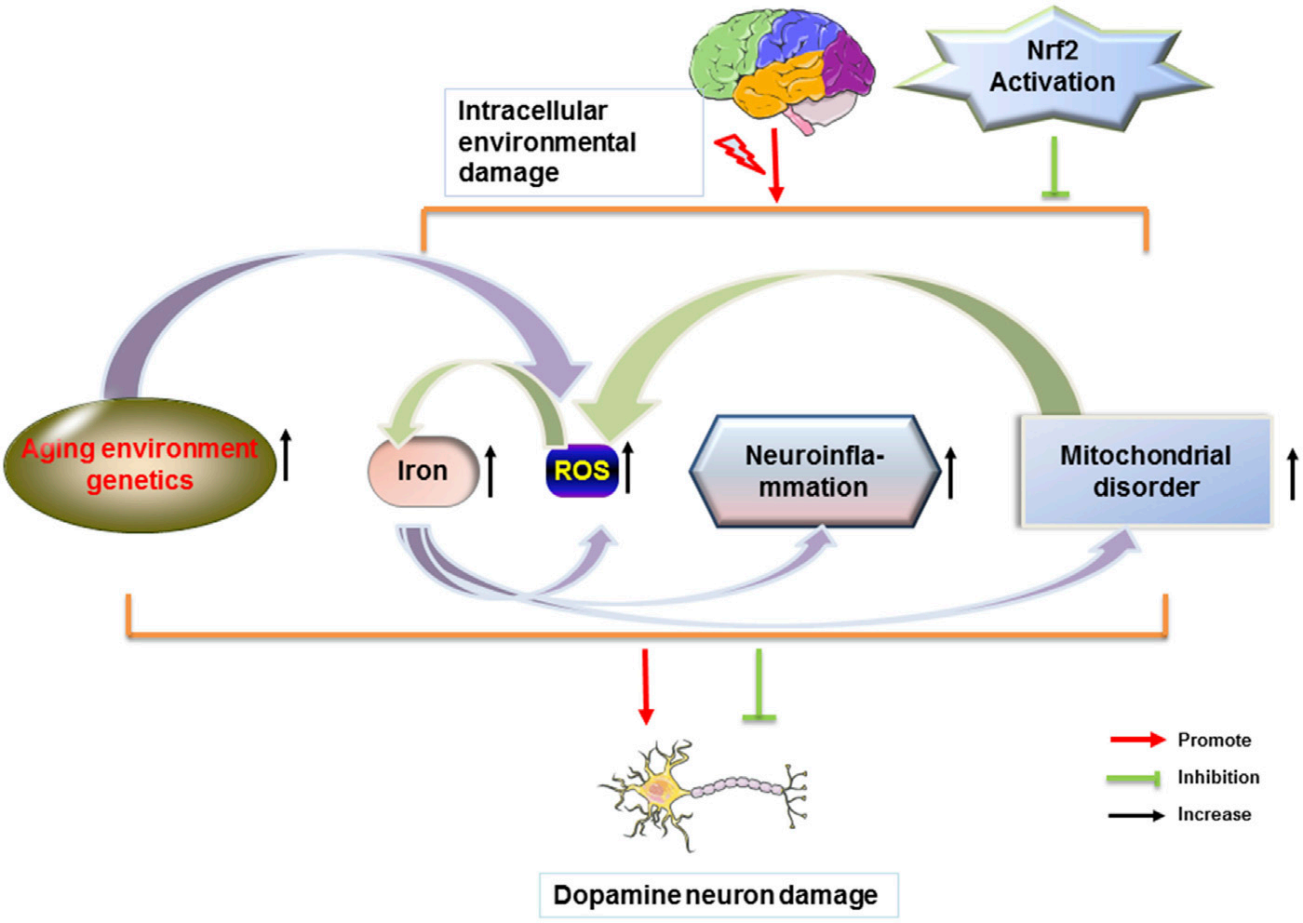
Nrf2 attenuates PD by modulating different factors. Dopamine neurons in the brain are disturbed by different factors. When homeostasis within the brain is dysregulated, a series of changes in the body, such as increased iron content in the brain, ROS production, glial cell-induced immune inflammation, and mitochondrial dysfunction occurs. Each factor can cause the loss of dopamine neurons in the brain and the misfolding of proteins to form Lewy body. Nrf2 reduces the loss of dopamine neurons and the formation of Lewy body and further improves PD.

The transcription factor Nrf2 has neuroprotective and anti-inflammatory effects and is an excellent molecular target for therapeutic agents related to PD. The physiological level of dopamine is beneficial to the organism. Too much dopamine could lead to the production of toxicants, which further cause oxidative stress and mitochondrial dysfunction. It has been shown that physiological concentrations of H_2_O_2_ are beneficial for dopamine neuronal survival by activating the Nrf2 signaling pathway in glial cells (Wang Y et al., 2021). The activation of the Nrf2 pathway allows a good protection of neuronal cells, which in PD is manifested by the reduction of cellular damage in oxidative stress and the improvement of PD symptoms. Synaptic nuclear proteins are the main components of Lewy’s body and an important feature of PD. α-synuclein is expressed in both *in vivo* and *in vitro* models of PD. α-synuclein aggregation is induced by downregulation of Nrf2 and Hmox1 (He et al., 2013). The increase in mutant synaptic nuclear proteins induces mitochondrial dysfunction and the elevated levels of ROS. Aggregation of activated Nrf2 to the nucleus reduces cellular damage from oxidative stress and ameliorates impairment of mitochondrial function, and also has modulatory effects on ions in the brain, neuroinflammation, and environmental genetics, thereby alleviating symptoms associated with PD (Figure 3).

### Nrf2 and Iron Death

Iron death as a new mechanism of cell death has been a hot topic of research in recent years. Iron death can affect the expression of many genes, and the mechanism of its role in PD is of great significance. The synthesis of ferritin heavy chains is dependent on the release of free iron and cytoplasmic iron efflux when the membrane transport protein Fe-ATPase is activated, leading to a decrease in intracellular free Fe^2+^ content and preventing the occurrence of Fentonian iron death-induced cell damage (Götz et al., 2004). Mitochondrial RNA (mtRNA) may alter iron death by regulating the expression of Nrf2 (Pérez-Soriano et al., 2020), where different isoforms have different regulation of Nrf2, inducing activation of Nrf2 pathway through inhibition of Keap1 or increasing the aggregation of Nrf2 in the nucleus through Cullin-3 (Cul3). Heme oxygenase (HO) is a protective factor, metabolite with Fe2+ production, and its Nrf2/HO-1 pathway can regulate intracellular iron concentration (LIP). However, whether it is the main pathway still needs further investigation. On the other hand, Nrf2 induction of solute carrier family 7 member 11 (Slc7a11) may affect the synthesis and function of GPx4, which is important in iron death (Song & Long, 2020). The activation of sequestosome 1(SQSTM1) /p62-Keap1-Nrf2- aldo-keto reductase 1C (AKR1C, metal binding protein MT-1G) pathway affects Nrf2 target genes HO-1 and NQO1 (Chorley et al., 2012) to regulate iron death. Nrf2 activation promotes iron aggregation in macrophages. In addition, microglia and astrocytes are involved in the regulation of iron homeostasis and reduce iron overload in DA neurons, demonstrating their protective properties.

In addition, activation of protein kinase C ζ (PKCζ) by ceramide (a brain derived neurotrophic factor signaling molecule) induces the casein kinase 2 (CK2)-Nrf2 signaling pathway, which protects dopamine neurons from iron death in the activated state. On the other hand, in a high iron-induced PD mouse model, the predicted role of Nrf2 in PD was found to show opposite results. In detail, Nrf2 knockout decreased intracerebral iron aggregation in the substantia nigra and striatum. It was inferred that Nrf2 knockout may reduce iron metabolism in the brain by downregulating iron transporter protein 1 (FPN1) expression levels in the brain and thus iron entry into the brain was inhibited (Han et al., 2021). The regulation of brain iron homeostasis by Nrf2 may be a potential therapeutic target for PD.

### Nrf2 and Oxidative Stress

Nrf2 is an important cellular defense mechanism involved in resistance to oxidative stress in PD. Cell is protected from oxidative stress by upregulating the differential expression of nearly 200 types of cytoprotective genes. Nrf2-Keap1 signaling pathway is a major defense system against oxidative stress and its induction of antioxidant enzyme gene expression allows cells to be well-protected (Zgorzynska et al., 2021). Generally, Keap1 is a negative regulator of Nrf2, and cysteine thiols enriched on Keap1 are oxidized by reactive substances, such as ROS, reactive nitrogen species (RNS), electrophile reagents, and xenobiotics, which increases the amount of nucleus by making Nrf2 appear unbound and regulates the redox state of the cell. Furthermore, Nrf2-associated AMP-activated protein kinase (AMPK) is phosphorylated in the cytoplasm and Nrf2 moves toward the nucleus upon its phosphorylation. Then, Nrf2 is phosphorylated and binds to sMaf protein. Nrf2 not only regulates redox homeostasis and mitochondrial biogenesis, but also upregulates antioxidant/electrophilic response elements (ARE/ERE), which can promote the expressions of protective genes (Goodfellow et al., 2020). Activation of Nrf2 signaling has made an obvious contribution to the discovery of drugs for the prevention and treatment of PD.

In addition, the main proteins regulated by Nrf2 are HO-1, nicotinamide adenine dinucleotide phosphate quinone reductase-1 (NQO-1), glutamate cysteine ligase, GSH, GPx, and several phase I and II enzymes (Jayaram & Krishnamurthy, 2021; Parga et al., 2021). These enzymes are closely associated with the production and consumption of ROS. Therefore, Nrf2 activation can effectively reduce dopamine neuronal damage caused by ROS. The damaging role of oxidative stress in PD and the protective role of Nrf2 in fighting free radicals let Nrf2 become a promising target for PD drug therapy. Currently, Nrf2 is of interest not only in PD but also in many other diseases affected by oxidative stress.

### Nrf2 and Neuroinflammation

Glial cells are confirmed to mediate neuroprotective effects. They usually express high levels of Nrf2 and Nrf2 pathway in astrocytes can be activated by dopamine to maintain the survival state of dopamine neurons. Nrf2 transcription factor is a key regulator of inflammation and has been verified to inhibit inflammatory responses induced by inflammatory factors, such as p65, IL-1β, TNF-α, caspase-1, and the nucletoide binding leucine rich repeat (NLR) family pyrin structural domain containing 3 (NLRP3) inflammasome. Moreover, NQO1 is a target gene downregulated by Nrf2, which inhibits the activation of NLRP3 inflammasome. Nrf2 contributes to the regulation of the effective anti-inflammatory HO-1 axis and the antioxidant gene HO-1 ultimately exhibits an anti-inflammatory function (Saha et al., 2020).

Recent evidence suggests that the the chemokine fractalkine (CX3CL1) /CX3CL1 receptor (CX3CR1) axis abrogates the deleterious effects on microglia proliferation in PD by upregulating Nrf2 expression. CX3CL1/CX3CR1 axis increases Nrf2-dependent gene expression *via* PI3K/AKT/glycogen synthase kinase 3 (GSK3) pathway (Subbarayan et al., 2022). In PD, CX3CL1 released by declining dopamine neurons binds to receptors expressed on microglia. Activation of Nrf2 signaling pathway prevents microglia overactivation. On the other hand, NF-κB induces the expression of various proinflammatory genes involved in the pathogenesis of inflammatory diseases (Cao et al., 2022). Nrf2 inhibits the activation of NF-κB signaling, which in turn plays a beneficial neuroprotective role in PD. In Nrf2 deficiency, inhibitors of NF-κB (IκB) are degraded by the proteasome to increase NF-κB. Genetically, DJ-1 prevents Keap1-mediated inactivation of Nrf2, stabilizes Nrf2, and increases the transcriptional activity of Nrf2. Nrf2 activates the transcriptional upregulation of PINK1 and protects dopamine neurons from oxidative stress-induced neurotoxicity.

### Nrf2 and Mitochondrial Function

Activation of Nrf2 upregulates the transcription of genes related to essential mitochondrial functions and increases the expression of the multifunctional DNA-binding protein mitochondrial transcription factor A (TFAM), which plays a key role in the expression of the mitochondrial genome. Mitochondria-targeted gene therapy is a potentially promising strategy to improve PD (Prasuhn & Brüggemann, 2021), and mitochondrial dysfunction was demonstrated in the PD mouse model.

In fact, nicotinamide adenine dinucleotide (NADH) and NADPH production from the mitochondrial tricarboxylic acid cycle and the cytoplasmic pentose phosphate pathway are essential for maintaining cellular redox homeostasis. Nrf2 could affect NADH and NADPH production through regulation of the pentose phosphate pathway. Also, Nrf2 controls the generation of ROS in mitochondria and NADPH oxidase. Normally, Nrf2 affects mitochondrial membrane potential involved in adenosine triphosphate (ATP) synthesis and respiratory function, various genetic and environmental factors can disrupt mitochondrial function, further leading to the accumulation of damaged mitochondria and increase oxidative stress. In the presence of oxidative stress or cytokines, Nrf2 can reduce ROS production (Bento-Pereira & Dinkova-Kostova, 2021). The tricarboxylic acid cycle of mitochondria and the pentose phosphate pathway of the cytoplasm provide access to redox-critical cofactors, such as NADH and NADHP, whose formation is closely linked to Nrf2. Among them, by regulating the pentose phosphate pathway, Nrf2 can be activated by tricarboxylic acid cycle intermediates (DeBlasi & DeNicola, 2020). Dysfunctional mitochondria leads to loss or absence of dopamine neurons due to untimely clearance of ROS, further leading to PD. Enzymes, genetic mutations, and ROS all also render mitochondria dysfunctional during this process, which could be regulated by Nrf2 and further improve PD symptoms.

### Nrf2 and Aging, Environment and Genetics

Compared to younger individuals, older animals and the elderly (>65 years) have lower Nrf2 nuclear content and less activation. The expression of Nrf2 transcription factors decreases with age in brain tissue, probably due to a decrease in the expression of Nrf2 target genes and an increase in NF-κB target genes, such as NQO1, γ-glutamylcysteine synthetase (γ-GCS), HO-1, intercellular adhesion molecule 1 (ICAM-1) and IL-6 (Silva-Palacios et al., 2018), which exacerbates the manifestations of PD. In addition, Nrf2-selective activators have been shown to prevent cellular senescence, such as rapamycin (Wang et al., 2017). On the other hand, environmental pollution can cause excessive activation of glial cells, oxidative stress, and neuroinflammation, which are closely related to neurodegenerative diseases, such as PD (Costa et al., 2020). As mentioned earlier, Nrf2 improved PD by regulating anti-inflammatory and antioxidant related proteins, such as Nrf2/HO-1, Nrf2/NF-κB, Nrf2/HO-1/NLRP3, Keap1/Nrf2, and Nrf2/HO-1/p38 (Han et al., 2022). Moreover, mutations in LRRK2 are the most common genetic factor in PD. Lovastatin, a β-hydroxy-β-methylglutaryl-coenzyme A (HMG-CoA) reductase inhibitor, activated Akt/Nrf2 pathway and inhibited the downstream gene GSK3β activity and further protected neurite degeneration in LRRK2-G2019S parkinsonism (Lin et al., 2016).

## Application of Nrf2 Activator on PD

The treatment of PD includes drug, surgery, and other treatments. The listed PD treatment drugs mainly contains catecholamine-O-methyltransferase inhibitor (COMTI) entacapone, anticholinergic drug phenylhexol, amantadine, monoamine oxidase type B inhibitor (MAO-BI) selegilan, dopamine receptor agonists, and compound levodopa (dopashydrazine and cardodopa). Levodopa is the best and effective standard symptomatic drug for PD. It highlights the importance of dopamine neurons and dopamine. Dopamine is an important neurotransmitter and plays a critical role in PD occurrence and development. The loss of dopamine neurons will undoubtedly induce the appearance of PD-related symptoms. At present, it is limited to supportive treatment that can partially alleviate PD symptoms, while disease eradication treatment is not feasible, and 85.1% of potential PD drugs fail in clinical trials (Boucherie et al., 2021). Nrf2-mediated antioxidant function has extraordinary significance in combating PD caused by the production of a large number of ROS. At present, various types of Nrf2 activators emerge in PD treatment, including electrophilic activators, such as sulforaphane (SFN), and Keap1-Nrf2 protein–protein interaction inhibitors.

### Modification of Keap1 Cysteine Residues by Nrf2 Covalent Activator

Toxic or electrophilic substances can activate Keap1-Nrf2-ARE signaling, and most Nrf2 activators are known to bind to the sulfhydryl group of cysteine residues in Keap1 through reactions, such as oxidation (Scuderi et al., 2020). Potential electrophilic activators or substances with electrophilic properties formed by metabolism interacted well with Nrf2-ARE and inhibited the ubiquitination of Nrf2 by reacting with Keap1 (cys273, cys151, and cys288) and then induced the expression of Nrf2-regulated cytoprotective factors. Dimethyl fumarate (DMF, tecfidera) and monomethyl fumarate (MMF, a metabolite of DMF) are found to attenuate neurotoxicity by covalently binding to the cys151 residue of the Keap1-BTB structural domain and further enhance Nrf2 activity. Moreover, sulforaphane (SFN) was discerned to prevent or slow down premature brain aging and memory loss in PD (Sunkaria et al., 2018). Dopamine neurons were protected by cys151 and cys171 residues of Keap1 as the key reactive sites reacting with SFN (Recio et al., 2018).

**FIGURE 4:**
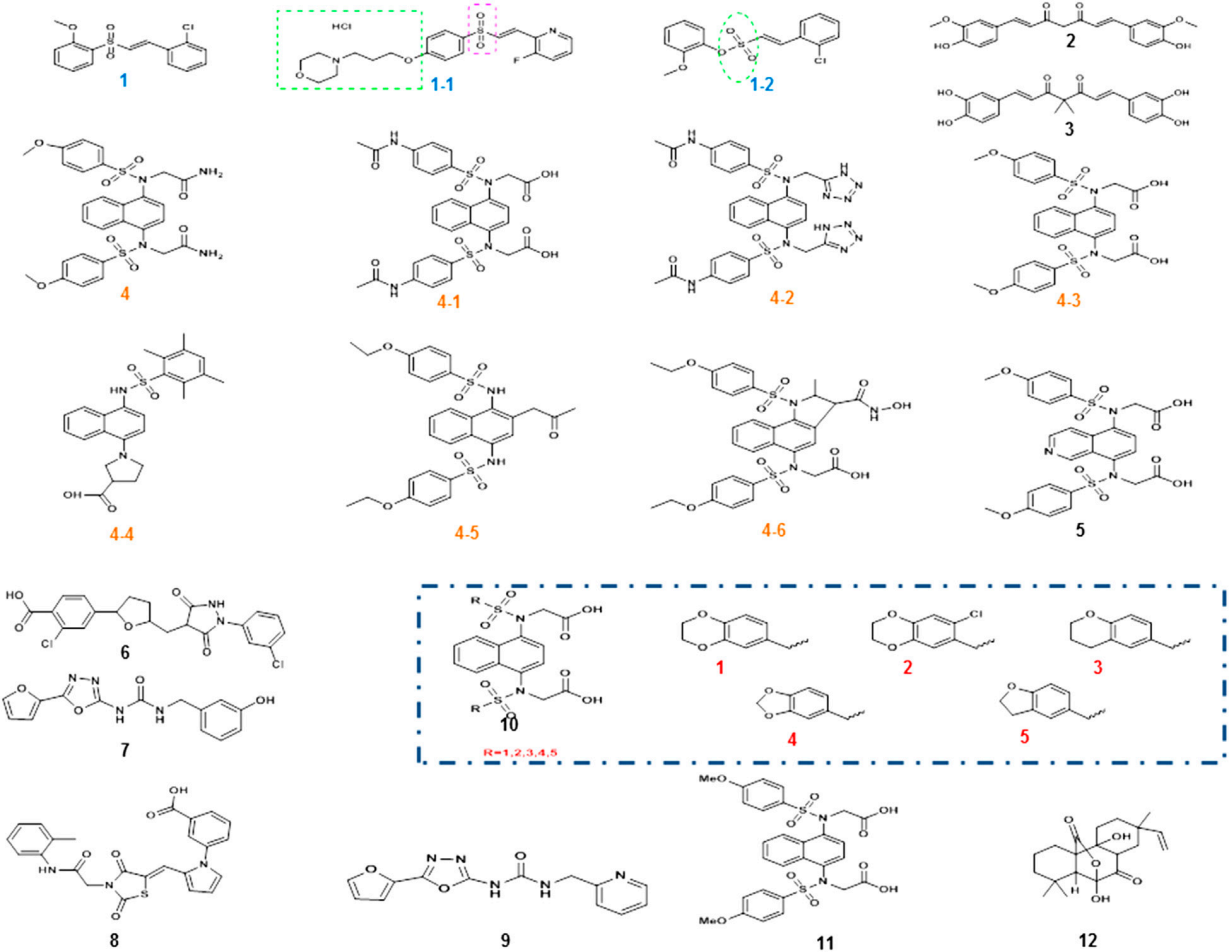
Chemical structures of Nrf2 activators and their analogs. Vinyl sulfone and its derivative compound 1, especially the more active compound 1-1, was obtained after the introduction of its nitrogen heterocycle, and the position of the nitrogen heterocycle was also indicated. Due to the specific toxicity of sulfone, sulfonamide was used instead of sulfone to obtain compound 1–2. The α, β-unsaturated carbonyl portion of curcumin compound 2 could bind to cys151 in Keap1 to prevent Nrf2 ubiquitination and proteasomal degradation. Modification of compound 2 structure yielded catechol derivative compound 3. Compound 4 is a small-molecule inhibitor of 1, 4-diaminonaphthalene nuclei, and a series of derivatives compound 4-1to 4-6 had similar activity, while other novel compound 8 (R = 1–5) and the compound 11 were also reported in PD. However, the aminonaphthalene core was not the best choice, giving rise to compound 5 with a 1, 4-isoquinoline core substitution, compound 7 with a 1, 3, 4-oxadiazole core structure, and compound 10 with an optimized structure. Two new structural compounds 6 and 9 were discovered and another series of PPI inhibitors containing five-membered heterocyclic and azole compounds were screened. Compound 12 was a potential activator of the Keap1-Nrf2-ARE pathway.

The Nrf2 activator vinyl sulfone, its derivative compound 1 (Figure 4), presented better efficacy against PD. The introduction of the nitrogen heterocycle yielded the more active compound 1–1 (Figure 4) with superior pharmacokinetics to compound 1 (Choi et al., 2019a). Considering that the physical and chemical properties of vinyl sulfone have several adverse effects on the organism, the substitution of vinyl sulfonamide or vinyl sulfonate is started from the aspect of vinyl sulfonate. In 2019, compound 1–2 (vinyl sulfonamide, Figure 4) was demonstrated to activate Nrf2 and then the antioxidant enzymes were enhanced and finally MPTP-induced PD mouse model was ameliorated (Choi et al., 2019b).

In addition, natural compounds are at the root of the prevention and treatment of PD. For example, curcumin compound 2 (Figure 4) and its derivatives have shown to hold antioxidant, anti-inflammatory, antiviral, antibacterial, antifungal, and anticancer properties (Liczbiński et al., 2020). Curcumin is able to penetrate the blood–brain barrier (BBB) and has been well studied in the protection against neurodegenerative diseases. Curcumin enhanced the activities of glutathione reductase, glutathione stransferase, and superoxide dismutase to confer cytoprotective effects *via* the activation of Nrf2 signaling. Further studies indicated that cys151 was a key target for curcumin modification of Keap1. The α, β-unsaturated carbonyl portion of curcumin binds and reacts with cys151 in Keap1 to prevent Nrf2 ubiquitination and proteasomal degradation. Its structure was modified to obtain catechol derivative compound 3 (Figure 4), whose bimethylation and catecholtype curcumin analogs were precursor molecules with cytoprotective activity, and the bimethylation and catechol-type curcumin analogs activated Nrf2 in michael receptor- and catechol-dependent pathways (Tu et al., 2017).

Similarly, the natural product obtusaquinone (OBT) was previously proved to have antitumor effects. Recently, the action of OBT and the characterization of its analogues were deep investigated. OBT could bind to cysteine residues of Keap1 with specific affinity and lead to Keap1 somatic degradation and activation of Nrf2 downstream expression factors. The ability of OBT was also confirmed to penetrate the protective barrier of the brain and work in the brain (Badr et al., 2020). The possible mechanism of action of OBT and its ability to penetrate the BBB greatly enhances its feasibility in studies related to PD prevention and treatment.

It is important to note that an increase in extracellular glutamate is a pathological change in PD. The increase in glutamate promotes oxidative stress in cells leading to cell damage and death. An interesting finding indicated that upon neuronal cells cultured using a small-molecule proteostasis regulator to prevent glutamate-induced cell death, the 2-amino-p-cresol substructure was required. Its resistance to glutamate-induced oxidative toxicity was also demonstrated in HT22 cells. Thus, a small-molecule proteostasis regulator covalently modified Keap1 to upregulate Nrf2-dependent resistance. Meanwhile, genes, such as ATF6 and the inositol-requiring enzyme 1 (IRE1)/X-box protein 1 (XBP1s), were activated *via* DESeq2 analysis of genes. Together, activation of Nrf2 in neurons by small protein homeostasis regulators involves selection of mechanisms for electrophilic and covalent modification of Keap1 (Rosarda et al., 2021).

### Direct Disruption of Keap1-Nrf2 Protein–Protein Interactions by Noncovalent Inhibitors

The Neh2 structural domain DLG and ETGE motifs of Nrf2 form distinct peptidomers with Keap1, revealing the importance of DLG and ETGE motifs in the ubiquitination and regulation of cellular Nrf2 activity (Canning et al., 2015) and peptide inhibitors exhibit high intrinsic affinity and low activity against Keap1. A novel cyclic peptide [c(GQLDPETGEFL)] with minimal acidic residues with glycine was obtained in a proper affixation method, revealing high affinity and activity toward Keap1-Nrf2 *in vitro* (Lu et al., 2018). The DEETGE amino acid sequence is located at amino acids 77–82 of Nrf2, which forms a peptide inhibitor with the Tat peptide and the calpain (Cal) break sequence (tat-cal-deetge). It can interfere with Nrf2-Keap1 interaction to activate Nrf2 *in vitro* and induce antioxidant gene expression in brain-injured mice (Zhao et al., 2011). Most peptides have not been studied clinically in neurodegenerative diseases due to the presence of BBB. Large molecules and modifications containing more hydrophilic groups did not work well.

Small-molecule protein–protein interactions (PPI) inhibitors act by a nonelectrophilic mechanism to directly disrupt Keap1-Nrf2 without the involvement of covalent bonds. Several substances with Keap1-Nrf2 PPI inhibitory activity were obtained by high-throughput screening. Compound 4 (Figure 4) is a small-molecule inhibitor containing 1, 4-diaminonaphthalene nuclei, and the activity of 1,4-diaminonaphthalene nuclei was confirmed by a series of derivatives (4-1 to 4-6, Figure 4) (Richardson et al., 2018). Recently, a series of novel 1, 4-bis (arylsulfonylamino) naphthalene-N, N′-diacetic acid analogues (compound 8 and 11, Figure 4) were calculated. Among them, compound 2 is well expressed in the cellular Nrf2 signaling pathway, which activates the expression of ARE-controlled Nrf2 target genes NQO1, HO-1, and proteins. The concern that aminonaphthalene core has cancer-inducing and mutagenic properties might be explained by the presence of the central naphthalene 13 scaffold. To improve the safety of Nrf2 activators, the non-naphthalene and heterocyclic scaffolds need further exploration.

Previous studies found high cellular activity and low mutagenicity of compound 5 (Figure 4) with a substituted 1, 4-isoquinoline core (Sun et al., 2014), and this finding provided the molecular backbone for the innovation of Keap1-Nrf2 PPI inhibitors. In addition, two new structural compounds 6 and 9 (Figure 4) were discovered and another series of PPI inhibitors containing five-membered heterocyclic and azole compounds were screened. By analyzing their binding to Keap1, compound 7 (Figure 4), which had the core structure of 1, 3, 4-oxadiazole, was found to bind effectively to Keap1. The optimized structure 10 (Figure 4) was obtained by performing SPR evaluation (Li et al., 2020). The high polarity of small-molecule inhibitors, which are highly limited in penetrating BBB, is mostly unsuitable for the treatment of PD. The discovery of a keap1-Nrf2 PPI interferer that is both highly active and readily crossing BBB is extremely challenging.

Recently, an isopimarane-type diterpenoid, sphaeropsidin A (SA, compound 12, Figure 4) was present. Its potency is about five times higher than that of sphaeropsidin (SF). SA is a good activator of the Keap1-Nrf2-ARE pathway, in which phosphatidylinositol-4, 5-bisphosphate 3-kinase (PI3K), protein kinase C (PKC), and protein kinase RNA-like endoplasmic reticulum kinase (PERK) are key enzymes of this pathway (Li et al., 2019). Its antioxidant properties are a beneficial resource for PD treatment.

Until now, Nrf2 activation has been confirmed to be protective against neurological diseases, and development of Keap1-Nrf2 PPI inhibitors might become a strategy for the treatment of PD. However, its large polarity is a challenge to overcome. Therefore, it is necessary to reduce the substitution of polar groups, and the design of targeted system delivery and predrugs would be further improved.

## Conclusion

The pathogenesis of PD is the result of multiple factors, and excessive ROS production is a common consequence of the process of necrotic apoptosis. However, its criticality in the mechanism of cell death is still under debate. This is because ROS are not essential for the induction of apoptosis in several cell types, such as the induction of cell necrosis by the cytokine TNF-α family, in which receptor interacting. Protein-3 (RIP3), a member of the RIP kinase family, is the determining factor (Zhang et al., 2017; Yang et al., 2018). Keap1-Nrf2-ARE is the main antioxidant pathway, and regulation of this pathway might control the development of PD. Nrf2 electrophilic activators entering clinical trials have led to the occurrence of adverse organismal reactions due to the nonselective and covalent modifications possessed by cells. Noncovalently bonded Nrf2 activators are extensively studied and have the promising potential of entering the clinic through preclinical studies.

So far, Nrf2 activators have not been used in the clinical treatment of PD. Therefore, more new compounds targeting Nrf2 need to be designed and characterized. In clinical applications, the ideal Nrf2 activator requires not only high efficiency, specificity, and stability, but also high bioavailability, good safety, and high BBB permeability. Lack of biomarkers in the early stage of PD motor characteristics makes PD patients difficult to receive early intervention. There is also a shortage of drugs that cannot cross BBB. Solutions to these challenges will await future studies in PD patients using novel antioxidant therapies that activate Nrf2.
